# Engineered recombinant protein products of the avian paramyxovirus type-1 nucleocapsid and phosphoprotein genes for serological diagnosis

**DOI:** 10.1186/s12985-018-0924-8

**Published:** 2018-01-11

**Authors:** Na Zhao, Christian Grund, Martin Beer, Timm C. Harder

**Affiliations:** The Federal Research Institute for Animal Health, Friedrich-Loeffler-Institut, Institute of Diagnostic Virology, Suedufer 10, 17493 Greifswald, Germany

**Keywords:** Newcastle disease virus, Recombinant protein, Subtype-specific serology

## Abstract

**Background:**

Virulent Newcastle disease virus (NDV, avian Avulavirus-1, APMV-1) induces a highly contagious and lethal systemic disease in gallinaceous poultry. APMV-1 antibody detection is used for surveillance and to control vaccination, but is hampered by cross-reactivity to other subtypes of avian Avulaviruses. Data are lacking concerning the applicability of NDV V proteins as differential diagnostic marker to distinguish vaccinated from virus-infected birds (DIVA strategy).

**Methods:**

Full length and C-terminally truncated nucleocapsid (NP) protein, and the unique C-terminal regions of the phospho- (P) and V proteins of the NDV LaSota strain were bacterially expressed as fusion proteins with the multimerization domain of the human C4 binding protein, and used as diagnostic antigens in indirect ELISA.

**Results:**

When used as diagnostic antigen in indirect ELISAs, recombinant full-length proved to be a sensitive target to detect seroconversion in chickens after APMV-1 vaccination and infection, but revealed some degree of cross reactivity with sera raised against other APMV subtypes. Cross reactivity was abolished but also sensitivity decreased when employing a C-terminal fragment of the NP of NDV as diagnostic antigen. Antibodies to the NDV V protein were mounted in poultry following NDV infection but also, albeit at lower rates and titers, after vaccination with attenuated NDV vaccines. V-specific seroconversion within the flock was incomplete and titers in individual bird transient.

**Conclusions:**

Indirect ELISA based on bacterially expressed recombinant full-length NP compared favorably with a commercial NDV ELISA based on whole virus antigen, but cross reactivity between the NP proteins of different APMV subtypes could compromise specificity. However, specificity increased when using a less conserved C-terminal fragment of NP instead. Moreover, a serological DIVA strategy built on the NDV V protein was not feasible due to reduced immunogenicity of the V protein and frequent use of live-attenuated NDV vaccines.

**Electronic supplementary material:**

The online version of this article (10.1186/s12985-018-0924-8) contains supplementary material, which is available to authorized users.

## Background

Newcastle Disease (ND) is caused by infection of chickens with virulent strains of the avian paramyxovirus serotype-1 (APMV-1, *syn*. ND virus, NDV). ND, together with highly pathogenic avian influenza, are among the most dreaded viral infections of gallinaceous poultry worldwide [1]. Rapid spread of a highly lethal, pantropic disease ensues in affected flocks after incursion of virulent (*syn*. Velogenic) NDV. Consequently, ND is an O.I.E.-notifiable disease of poultry. The virus is enzootic in many regions of the world and continues to threaten industrial production as well as backyard poultry holdings. Vaccination with inactivated adjuvanted or, alternatively, live virus vaccines featuring attenuated (lentogenic) APMV-1 strains such as LaSota or B1 is widely used to prevent ND [2, 3].

Avian avulaviruses are members of the *Avulavirus* genus within the *Paramyxoviridae* family. To date at least fifteen serotypes (APMV-1 to APMV-15) have been identified [4–11]. The virus possesses a single stranded, negative-sense, non-segmented RNA genome, which encodes six structural proteins in following order: nucleocapsid protein (NP), phosphoprotein (P), matrix protein (M), fusion protein (F), hemagglutinin-neuraminidase protein (HN), and the large (L) polymerase protein [12, 13]. The NP protein is a highly conserved and the most abundant viral protein expressed in infected cells, and induces a strong humoral (non-neutralizing) and cellular immune response in the infected host and also following vaccination with inactivated virus [3, 14–16].

The P protein plays an important role in the viral transcription and replication, and it is associated with the nucleocapsid in the virion [17, 18]. Two additional proteins, V and putative W, are predicted to be produced from P gene by mRNA editing post transcription [19–23]. The product that ensues by insertion into the nascent P mRNA of one non-template G residue at position 401 (+2 reading frame) is referred to as the V protein [21]. Addition of two untemplated G’s at the polymerase slipping point of the P gene would generate a third protein species, the W protein, from the P gene [21, 23]. Thus, all three P gene-derived proteins have a common N-terminus, but vary at their carboxyl termini both in length and amino acid composition. The V protein of NDV harbors 106 amino acids in its unique C-terminal part (LaSota strain). Similar to other viruses in the *paramyxoviridae* family, the V protein is found to be a zinc-finger domain protein and appears to function as a virulence factor by antagonizing, in a strain-specific manner, components of the host innate immunity, in particular the interferon system [24, 25]. However, very little is known about the immunogenicity of the V protein.

Serological assays to detect ND-specific antibodies can be used for demonstration of lack of exposure of a flock to NDV, and for assessment of vaccination efficacy. The hemagglutination inhibition (HI) test is a standard method and widely used for NDV antibody detection, although it may lack in sensitivity and is time-consuming [26–28]. Several ELISA formats have been developed as an alternative for conventional HI test in flock screening approaches. Their sensitivity, and easy standardization make them suitable for high throughput screening [28–34]. ELISA formats based on whole virus and recombinant viral proteins expressed in baculovirus or bacterial systems as the coating antigen have been reported [28, 29, 35–38]. Recombinant NP protein in particular has been used for the development of indirect ELISAs (iELISA) [14, 35]. However, the NP protein is highly conserved among avian paramyxoviruses, and serologic assays building on recombinant NDV NP may be compromised by cross reactions between various APMV serotypes.

To overcome limitations of full length NDV NP as the antigen in ELISA format, we hypothesized that use of less conserved NP and P protein fragments would enable a more serotype-specific distinction. The objective of this study was to explore the suitability of truncated NP and P proteins as diagnostic antigens for detection and differentiation of avian paramyxovirus-specific antibodies. Moreover, assuming that a humoral immune response against the V protein would be enhanced by active viral replication in the host and expression of V in infected cells, V protein-specific antibodies might be used to distinguish infected birds from those that are seropositive after vaccination with inactivated or attenuated vaccines. Therefore, another objective was to explore and evaluate the diagnostic feasibility of using the unique portion of V protein in an indirect ELISA-based DIVA (differentiating infected from vaccinated animals) strategy.

## Methods

### Virus propagation

APMV-1 vaccine strain LaSota (LS; class II, genotype 2) [39] and APMV-8 strain goose/Delaware/1053/76 were grown in the allantoic cavity of embryonated chicken eggs as detailed elsewhere [28].

### Cloning and bacterial expression of recombinant proteins

**Fig. 1:**
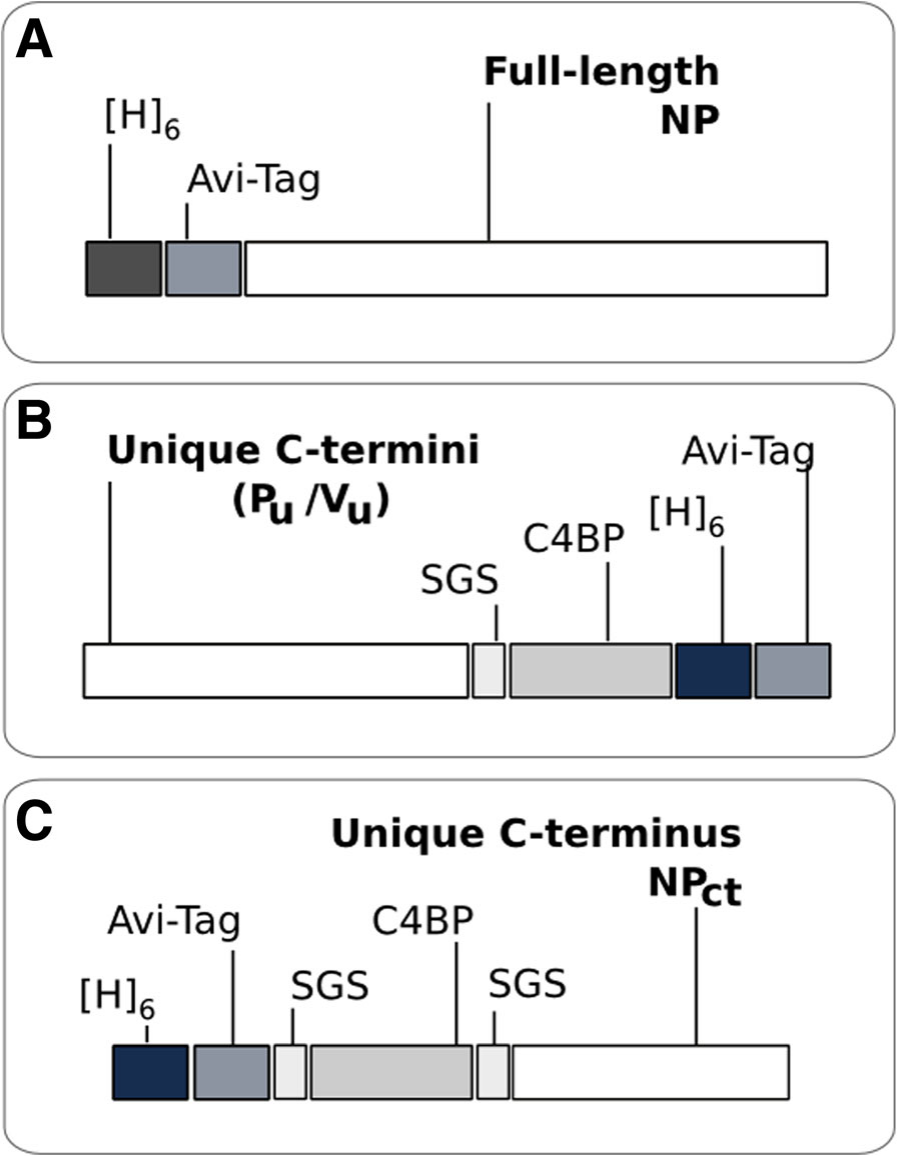
Schematic presentation of constructs used for generation of pET19b plasmids and T7- driven expression of NDV proteins in Rosettagami *E. coli* cultures. All sketches show the protein C-terminus. **a** Design of full-length expression of the nucleocapsid gene of NDV strain LaSota, with N-terminal hexahistidin ([H] 6) and AVI tags. **b** Expression of truncated and frame-edited versions of the P gene expressing unique C-termini of the two P gene-derived proteins P and V as fusion proteins with the heptamerization domain of the human C4 binding protein (C4BP), and tags. An SGS-linker separates virus-specific from fused protein sequences. **c** Expression cassette of C-terminally truncated versions of the NP protein of NDV LaSota or APMV-8, respectively

The pET19b vector and Rosettagami *E. coli* cells (both Novagen, Darmstadt, Germany) were used for expression of recombinant NDV proteins. The full length ORF encoding the nucleocapsid protein (NP) of NDV LaSota was cloned into pET19b downstream of the T7 promoter as shown in Fig. 1a. A hexa-histidine- and an Avi-tag were positioned in-frame and N-terminally of the ORF [40]. Similarly, the sequences encoding the unique parts of the P and V (P_u_, V_u_) downstream of the RNA editing site of the P gene were cloned into pET19b (Fig. 1b). In addition, the C-terminal 99 amino acids of NDV LaSota NP (NP_ct_), and the C-terminal 81 amino acids of the NP of APMV-8 were cloned as shown in Fig. 1c. A heptamerization fragment of the human C4 binding protein (C4BP) was positioned between the tags and the NDV sequences [41, 42]. SGS-linker sequences were placed between C4BP and the virus-specific sequences to ensure unrestrained folding of the latter. Plasmid pBirCam (Avidity, Aurora, U.S.A) over-expressing the bacterial biotin ligase BirA was co-transformed into strain Rosettagami to ensure cotranslational mono-biotinylation of the recombinant APMV protein fragments at their Avi-tag [43]. Ampicillin and chloramphenicol were used for selection. Presence of both plasmids was confirmed by plasmid-insert-specific PCRs.

Induction of expression was achieved in TYH medium supplemented with IPTG (1.5 mM) and D (+)-biotin (50 μM). After culturing for 4 h at 37 °C, cells were pelleted and lysed using ultrasonic disruption as previously described [43] to liberate inclusion bodies (ICs) into which recombinant proteins had sequestered.

### Purification and reconstitution of recombinant proteins from bacterial inclusion bodies

Purified ICs were solubilized in 6 M guanidinium-HCl. A set of refolding buffers was used in a stepwise solubilisation strategy using the ProteoStat kit (Enzo, Lörrach, Germany) to determine the most appropriate buffer conditions to fold back the proteins into an antigenically authentic structure while keeping it solubilized. Antigenic properties in ELISA were assayed using polyclonal antibodies raised in chickens. Final protein concentration in refolding buffer was determined using a coomassie protein assay kit (ThermoScientific, Rockford, IL, U.S.A.) and proteins were stored at 4 °C until further use.

### Production of antisera

Galline hyperimmune sera against reference isolates of APMV serotype-1(NDV strain Ulster, pigeon paramyxovirus, class I isolate APMV-1/Mallard/Germany/R2481/2007), and other serotypes-2 (APMV-2/chicken/California/Yucaipa/56), −3a (APMV-3/turkey/England/1087/82), −3b (APMV-3/Parakeet/Netherlands/449/75), −4 (APMV-4/duck/Hong Kong/D3/75), −6 (APMV-6/Dk/HK/77), −7 (APMV-7/dove/Tennessee/4/75), −8 (APMV-8/goose/Delaware/1053/46), and −9 (APMV-9/duck/New York/22/78) had been generated in chickens by booster immunization using egg-grown virus adjuvant with incomplete Freund’s adjuvant. SPF chicken sera were sampled from chickens hatched at the FLI from SPF eggs (Lohmann Tierzucht, Cuxhaven, Germany). All animal experiments had received full legal approval by the animal welfare committee of the German Federal State of Mecklenburg-Vorpommern (LALLF M-V/TSD/7221.3–2.5-004/10; LALLF M-V/TSD/7221.3–2.5-010/10).

### Western blotting

Protein samples were separated by SDS-PAGE in 12.5% gels. For denaturing conditions samples were heated in Laemmli loading buffer containing dithioerythritol (DTE). Proteins were also separated under non-denaturing conditions in 10% PAGE without SDS and DTE. Proteins were transferred onto nitrocellulose membranes by semi-dry blotting at 0.8 mA/cm^2^ for 1 h (PerfectBlueTM Electro Blotter, Peqlab, Erlangen, Germany). Membranes were blocked with 5% skim milk powder in Tris-buffered saline (TBS) supplemented with 0.05% (*v*/v) Tween (TBST) for 1 h. Specific antibodies and sera were appropriately diluted in TBST and incubated on the membranes for 1 h at room temperature. Membranes were washed three times with TBST and incubated for another hour with POD-labeled secondary antibody. Blots were washed again for three times, then incubated with substrate SuperSignal™ West Pico Chemiluminescent substrate solution (ThermoScientific, Braunschweig, Germany) before being analyzed with an imaging system (VersaDoc, Bio-Rad). Photos were edited with respect to contrast and brightness and composite blots were assembled using cut-out lanes (GIMP software).

### Indirect ELISA

Recombinant proteins solubilized in the respective refolding buffers were further diluted to a concentration of 5 μg/ml using bicarbonate coating buffer (pH 9.6) of which 100 μl per well were used to coat Maxisorb ELISA plates (NUNC, Thermofisher, Braunschweig, Germany) overnight at 4 °C. After washing, wells were blocked by 5% skim milk-TBST) for 2 h at room temperature and then washed four times with TBST [44]. Individual sera were diluted 1:200 (for the NP iELISA) or 1:500 (other antigens) in appropriate sample dilution buffer. Sera were incubated at room temperature for 1 h. Wells were washed again four times with TBST before 100 μl of appropriately diluted goat-anti-chicken or -turkey IgY POD conjugate (Dianova, Hamburg, Germany) was added for 1 h at room temperature. Antibody was removed and after a final washing cycle with TBST, 50 μl of chromogenic TMB substrate was added. OD450 values were measured after 10 min of incubation and addition of 50 μl of 1 N H_2_SO_4_ to each well. Results were calculated and expressed in S/P units:

$$ \frac{\mathrm{ODTest}\hbox{-} \mathrm{ODBackground}}{\mathrm{ODPositive}\kern0.5em \mathrm{control}\hbox{-} \mathrm{ODBackground}}\times 100=\mathrm{S}/\mathrm{P} $$

### Determination of cut-off values for indirect ELISAs

**Fig. 2:**
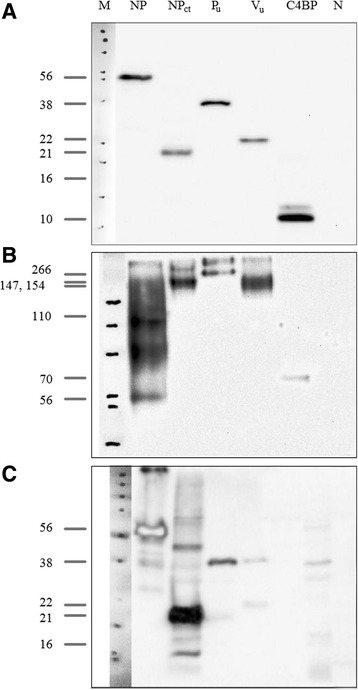
Assembled Western blot assays of monobiotinylated recombinant proteins of NDV LaSota. Panels **a** and **b** were stained using a monoclonal antibody specific for biotin. For panel **c** a chicken serum (S880/Hu295) was used that was obtained at day 28 after immunization with live-attenuated NDV LaSota vaccine. Panels **a** and **c** were run under denaturing conditions while panel **b** was obtained after protein separation in native PAGE. M – marker lane, NP – full length nucleocapsid protein; NP_ct_ -C-terminal fragment of nucleocapsid protein; P_u_ – unique C-terminal part of phosphoprotein; V_u_ – unique C-terminal part of V protein; C4BP – heptamerization domain of human C4 binding protein; N – Rosettagami *E. coli* lysate. Molecular weights of monomeric recombinant proteins (panels **a**, **c**) and multimeric conglomerates (panel **b**) are presented to the left of the marker lane

Maxisorb ELISA plates were coated with 0.5 μg of recombinant protein per well and then blocked. A predilution step of sera, as outlined above, was required for background reduction. Species-specific anti-IgY POD-conjugates were used for detection of bound antibodies. A total of 51 sera from SPF chickens were examined to determine cut-off values for the different recombinant antigens in this indirect ELISA format. None of these sera tested positive in a commercial indirect NDV ELISA (see below). Mean extinctions and standard deviations were calculated (see Additional file 1). Serum S185, a hyperimmune serum raised in chicken against inactivated NDV strain Ulster, served as standard positive control for assays employing NP and P antigens. Serum S880/Hu295 was used as standard positive control for V protein-specific assays since this serum had been obtained at day 28 after immunization with live-attenuated NDV LaSota vaccine and was shown to harbor V-specific antibodies in Western blotting (Fig. 2c). An SPF chicken serum was chosen as standard negative control. These control sera were used as standards in each of the assays to determine threshold OD values and to calculate S/P ratios. The S/P mean values of all 51 SPF sera plus 2 SD were chosen as cut-offs in the examination of chicken field sera by indirect ELISAs. Additional file 1 presents exact cut-off values for each assay.

### Commercial ELISA for detection of NDV antibodies

A commercial indirect NDV-ELISA (Flock Chek NDV) based on complete ND virions was purchased from IDEXX (Ludwigsburg, Germany). Instructions of the manufacturer were followed exactly using either the version for chickens or turkeys as appropriate.

### Hemagglutination inhibition assay (HI)

HI assays were performed according to O.I.E. recommendations essentially as described [45]. Four hemagglutinating units of egg-grown APMV-1 or APMV-8 viruses were used throughout. All sera had been heat-inactivated for 30 min at 56 °C.

### Origin of field sera

A total of 150 chicken and 147 turkey sera submitted for routine avian influenza or ND serodiagnostic investigations originated from various poultry holdings in Germany. It should be noted that, in Germany, NDV vaccination is compulsory for all gallinaceous poultry including those kept in small backyard flocks.

### Origin of experimental infection sera

#### APMV-1

A total of 10 chickens hatched from eggs of NDV vaccinated hens received immunization on day 14 post hatch applying live-attenuated vaccine based on NDV strain “Clone 30” (Table 1, vaccinated group). An additional six hatch mates were used as controls and did not receive vaccination. At day 29 post vaccination i.e. day 43 post hatch all chickens were challenged with the velogenic NDV strain “Herts 33/56” and serum samples were taken at days 0, 3, 7, 14, 21, and 28 post challenge.

**Table 1:** Serology of chickens challenged with velogenic NDV strain Herts following vaccination with live-attenuated NDV vaccine

DPC	Group	Serological assays				
		Commercial NDV iELISA	HI	iELISA-NP_ct_	iELISA-P_u_	iELISA-V_u_
0 DPC	vaccinated	8/10^1^	8/10	9/10	9/10	2/10
	control	0/6	0/6	0/6	0/6	0/6
3 DPC	vaccinated	9/10	9/10	9/10	7/10	2/10
	control	0/6	0/6	0/6	0/6	0/6
7 DPC	vaccinated	10/10	10/10	10/10	8/10	7/10
	control	4/4	4/4	4/4	2/4	2/4
14 DPC	vaccinated	10/10	10/10	10/10	8/10	7/10
	control	4/4	4/4	4/4	4/4	4/4
21 DPC	vaccinated	9/9	8/9	9/9	8/9	7/9
	control	4/4	4/4	4/4	4/4	4/4
28 DPC	vaccinated	6/6	5/6	6/6	4/6	4/6
	control	4/4	4/4	4/4	4/4	2/4

Vaccinated group – Ten chickens each were vaccinated with NDV strain “Clone 30”

Control group – Six chickens did not receive vaccination before challenge

DPC – days postchallenge; challenge infection with NDV strain Herts33 was carried out on day 21 post vaccination, i.e. day 0 DPC characterizes the status at day 21 post vaccination

HI – hemagglutination inhibition assay against NDV LaSota antigen

^1^N/M – numbers of seropositive/total chickens

#### APMV-8

Fifteen chickens were immunized with wild-type APMV-8 strain APMV-8/goose/Delaware/1053/76 (GenBank acc. no. FJ619036) one day after hatch and challenged with APMV-8 on day 21 post vaccination. as described in detail by Grund et al. [46]. Samples used here for serological analyzes were taken on days 21 post vaccination and 14 post challenge. Both animal experiments received full legal approval by the animal welfare committee of the Federal State of Mecklenburg-Vorpommern (LALLF M-V/TSD/7221.3–1.1-053/10).

## Results

### Production of recombinant proteins

Recombinant proteins were designed to represent those amino acid sequences of the APMV-1 P gene products which are unique (“u”) to P and V. Since this resulted, especially for V, in comparatively small peptides (P_u_ 27.6 kD; V_u_ 11.7 kD), the heptamerization domain of the human C4 binding protein (C4BP) was fused as a carrier module in order to facilitate handling as well as proper presentation of the specific peptide antigens (Fig. 1). The same strategy was used to express the C-terminal 99 amino acids of the LaSota NP protein (LS-NP_ct_) of APMV-1 which is much less conserved among APMV serotypes compared to the full-length NP protein (see Additional file 2). Also, the NP_ct_ of an APMV-8 strain was expressed as an example of a non-NDV APMV serotype. In addition, full length APMV-1 NP was expressed but without the C4BP fusion. All constructs harbored hexahistidine and AVI tags [47] to facilitate downstream processing (Fig. 1). The AVI tag was used for co-translational mono-biotinylation in *E. coli* in which BirA, a biotin ligase, was overexpressed.

Probing refolded recombinant proteins by Western blot analysis under reducing conditions with a monoclonal antibody against biotin yielded specific bands in the size range calculated from the amino acid sequence for NDV full-length NP as well as LS-NP_ct_, P_u_, V_u_ (Fig. 2a). The Rosettagami *E.coli* lysate and recombinant heptamerization domain of the human C4 binding protein expressed without any APMV-specific peptides were used as controls (Fig. 2a, lanes “N and C4BP”). When separating under non-denaturing conditions, proteins that contained the C4BP domain assembled into multimeric complexes. Their molecular weight measured under native PAGE conditions were approximately sevenfold the calculated value of their corresponding monomeric protein (Fig. 2b, lane “NP_ct_, P_u_, V_u_ and C4BP”). This indicated functionality of the heptamerization domain of C4BP. Intriguingly, apart from the monomers, also dimers and oligomers of the full-length NP protein were observed as well (Fig. 2b, lane “NP”) demonstrating that the refolded full-length NP has the ability of spontaneously forming multimers without the C4BP multimerization domain helper.

Immunogenicity of the APMV-specific peptide part was confirmed, by a chicken serum (S880/Hu295) obtained at day 28 after immunization with live-attenuated NDV LaSota vaccine. Strong responses to full length NDV NP, NP_ct_ and P_u_ as well as weak reactivity to V_u_ protein, visible as faint band at the expected size, proved that the major epitopes were preserved and accessible in the recombinant proteins after the refolding procedure (Fig. 2c). The results indicate that expressed recombinant proteins constitute suitable antigens for testing NDV specific antibodies in avian sera.

### Use of recombinant NDV proteins in indirect ELISAs (iELISA)

Indirect ELISA assays for all four recombinant NDV-antigens and an APMV-8 NP_ct_ antigen were established, applying a total of 51 sera from SPF chickens to determine cut-off values (see Additional file 1). A defined positive serum (S880/Hu295) and a selected SPF-chicken serum served as standards in each of the assays in order to determine validity (threshold OD values) and to calculate S/P ratios.

### Analytical specificity of recombinant NDV NP- and P-gene derived proteins in iELISA with sera raised against different avian avulavirus subtypes

**Fig. 3:**
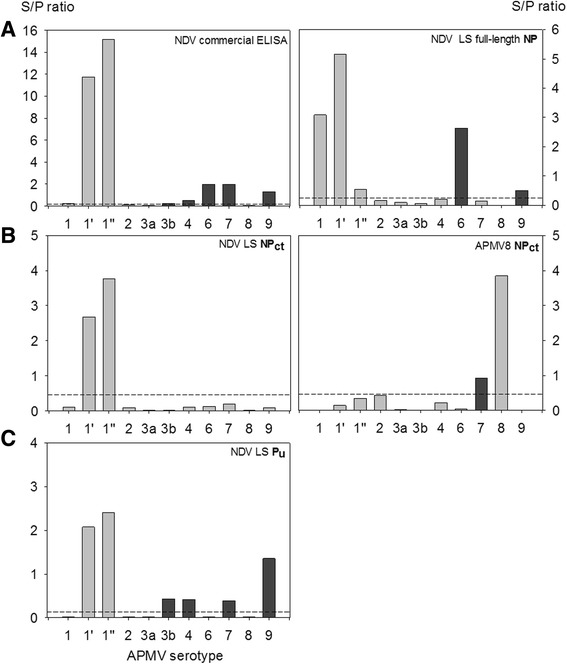
Use of NP and P gene products of APMV-1 and APMV-8 (NP) as diagnostic antigens in indirect ELISA compared to a commercial NDV ELISA. NDV commercial iELISA and Full length NP of NDV strain LaSota (NDV LS full-length NP) iELISA were showed in panel **a**; C-terminal fragment of the NDV (NDV LS NP_ct_) and the APMV-8 (APMV-8 NP_ct_) NP protein based iELISA were presented in panel **b**; The truncated P (NDV LS P_u_) iELISA were in the panel **c**. The chicken hyperimmune sera raised against avian avulavirus subtypes 1 through 9. 1 – APMV-1 class I, 1′ – APMV-1 class II, LaSota, 1″ – APMV-1 class II, pigeon paramyxovirus, other serotypes as indicated. Dotted line indicates cut-off. Dark grey bars indicate unspecific reactivity

**Fig. 4:**
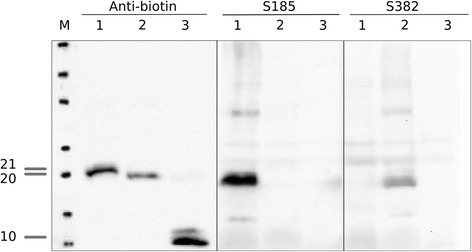
Assembled Western blot assays of monobiotinylated recombinant NP proteins (C-terminal fragments) of APMV-1 (NDV LaSota, lane 1, 21 kD), APMV-8 (lane 2, 20 kD), and the heptamerization domain of the C4 binding protein (lane 3, 10 kD). Anti-biotin – monoclonal antibody directed against biotin; S185 – hyperimmune serum raised in chicken against NDV strain Ulster; S382 –hyperimmune serum raised in chicken against APMV-8 strain/ goose/Delaware/1053/76. Molecular weights (kD) are indicated to the left

Recombinant iELISAs were used to test chicken hyperimmune sera raised against inactivated antigens of nine APMV serotypes; for APMV-1 and APMV-3 sera were raisted against each of 3 (APMV-1 class II/Ulster, PPMV, APMV-1 class I) and 2 (APMV-3/England; APMV-3/Netherlands) strains, respectively. Other sera were raised against APMV-2, −4, −6, −7, −8, −9. A commercial NDV ELISA based on virion-preparations as antigen was used as a control. In this ELISA, only 2 out of 3 APMV-1 sera reacted (Fig. 3a) but a hyperimmune serum raised against APMV-1 class I was not detected. In addition, APMV-6, 7 and 9 hyperimmune sera showed clear cross reactivity (dark greyed columns in Fig. 3), whereas the APMV-4 hyperimmune serum revealed marginal reactivity, but was considered APMV-1 positive by internal standards of the commercial ELISA. When using recombinant full length LaSota NP protein, positive reactions were obtained with all APMV-1 sera, including the APMV-1 class I serum. However, cross reactivity of APMV-6 and -9 specific sera remained (Fig. 3a). In the NDV NP_ct_ based iELISA, in contrast, only NDV-specific sera raised against APMV-1 class II viruses (NDV LaSota, and the pigeon paramyxovirus type-1) reacted while all cross reactions were abolished (Fig. 3b, left panel). No cross reactivity was observed with a serum specific for APMV-1 of class I. Conversely, by the APMV-8 NP_ct_ iELISA, the corresponding homologous antiserum was detected while cross reactions were limited to an APMV-7 serum only. Serotype specificity of NP_ct_ antigens to corresponding APMV-1 and APMV-8 hyperimmune sera was also confirmed in composite Western blot assays (Fig. 4).

Similar to the recombinant full-length NP iELISA, cross reactivity of serotype-specific APMV sera was observed also for the iELISA when using the P_u_fragment as the coating protein: Sera specific for APMV-3b (Netherlands), −4, −7, and −9 revealed reactivity above threshold level against the LaSota P_u_ protein (Fig. 3c).

### Detection of NDV NP and P antibodies in vaccinated/challenged chickens

Ten chickens were immunized with a live-attenuated vaccine based on NDV strain “Clone 30” [48]. Six SPF chickens were used as controls. At day 21 post vaccination, all chickens were challenged with the velogenic NDV strain “Herts” and serum samples of surviving chickens were taken at days 0 (= day 21 post vaccination), 3, 7, 14, 21, and 28 post challenge(p.c.). Results obtained with these sera in iELISAs using recombinant NP_ct_ and P_u_ proteins were compared to commercial NDV ELISA and to HI titers against the LaSota strain of NDV. HI titers higher than 1:8 were considered positive.

**Fig. 5:**
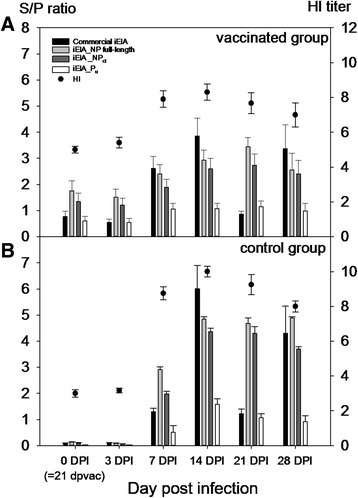
Antibody kinetics in NDV vaccinated and challenged chickens against APMV-1 measured by different ELISA formats and HI assay. Sera were obtained from ten chickens after immunization with a live attenuated vaccine based on NDV strain “Clone 30” and challenge at day 21 post vaccination (= day 0 post infection) with the velogenic NDV strain “Herts” (panel A). Panel B shows kinetics of six chickens that were not vaccinated before challenge. Antibodies were measured by indirect ELISAs as indicated in the figure legend or by HI assay against the NDV LaSota strain antigen

As shown in Table 1, NDV antibodies were detected in a majority of the vaccinated chickens at day 0 post challenge by HI, commercial ELISA and indirect NP_ct_ ELISA assays, whereas a lower number of birds were positive by the P_u_ ELISA assay. Moreover, in the unvaccinated control group, all chicken sera tested negative at day 0 p.c. and had seroconverted until 7 days p.c. in HI, commercial ELISA and indirect NP_ct_ ELISA assays, but complete seroconversion of the control group to P_u_ was delayed to day 14 p.c. (Table 1). This indicates that the iELISA based on the P_u_ protein is less sensitive than the other assays. The development of antibody titers as measured by the different ELISAs and compared to HI titers of the vaccinated and control groups before and after challenge is shown in Fig. 5.

When testing sera from fifteen chickens immunized with wild-type APMV-8 strain, 14 out of 15 serum samples were positive in the APMV-8 NP_ct_ iELISA. No cross reactivity was seen with the APMV-1 NP_ct_ based iELISA assay (Table 2).

**Table 2:** Qualitative results of serological investigations (seropositive/total samples analysed per assay)

serum panel	HI assay		Commercial ELISA	Recombinant ELISA			
	NDV LS	APMV-8		NP full-length	P_u_	LS NP_ct_	APMV-8 NP_ct_
SPF	0/51	0/51	0/51	0/51	0/51	0/51	0/51
Experimental Infection NDV^a^	68/83	n.d.	68/83	69/83	58/83	68/83	0/83
Experimental Infection APMV-8^b^	0/15	15/15	0/15	0/15	n.d.	0/15	14/15
Field sera chicken	60/150	n.d.	103/150	91/150	90/150	79/150	0/150
Field sera turkey	67/147	n.d.	113/147	100/147	57/147	88/147	0/147

^a^Experimental NDV infection sera were obtained from chickens after immunization with a live attenuated vaccine based on NDV strain “Clone 30”; on day 21 post vaccination all chickens were challenged with thes velogenic NDV strain “Herts”

^b^Experimental APMV-8 infection sera originated from chickens that were immunized with wildtype APMV-8 strain APMV-8/goose/Delaware/1053/76 one day after hatch and challenged with APMV-8 on day 21 postvaccination. Samples used here for serological analyzes were taken on days 21 post vaccination and 14 post challenge

### Antibody response to V_u_ protein in chickens vaccinated with live-attenuated NDV and challenged with a velogenic NDV strain

The same set of sera as described in the previous section was examined for V protein specific antibodies in the V_u_ iELISA. Before challenge with the velogenic NDV strain, the majority of vaccinated chickens had seroconverted by HI and commercial ELISA tests, but only two birds reacted in the V_u_ protein assay (Table 1). Following challenge, V_u_-specific seroconversion was evident from day 7 p.c. onwards and a maximum of seven out of 10 of the challenged vaccines were positive. Additionally, V_u_protein specific antibody was detectable in all four unvaccinated control chickens from day 14 p.c. onwards. V_u_-specific antibody titers declined in vaccinated and in control birds at day 28 p.c. (Table 1).

### Detection of NDV-specific antibodies in chicken and turkey field sera

Finally, 297 field sera from NDV vaccinated chickens (*n* = 150) and turkeys (*n* = 147) were tested in a commercial indirect NDV ELISA, and by HI, and results were compared to data obtained with iELISAs using recombinant proteins (Table 2). There was high correlation between the commercial ELISA and the results obtained with recombinant full-length NP (Kappa = 0.688 [95% CI 0.604–0.773]) while both assays only had moderate correlation with the HI assay using antigen of the NDV LaSota strain (commercial ELISA: Kappa = 0.401; recombinant NP iELISA: Kappa = 0.458). This was caused by a considerable proportion of sera which reacted positive in either of the ELISAs but remained negative by HI.

Results from APMV-1 NP_ct_ based iELISA yielded less numbers of positive sera compared to both full-length NP and commercial ELISA assays (Table 2). Comparison of results obtained with the commercial NDV ELISA and the two recombinant NDV NP antigens showed good agreement of the three assays (Venn diagram, Fig. 5) although the NP_ct_ ELISA revealed reduced sensitivity. Including results obtained with the SPF sera and the experimental NDV infection sera, a sensitivity of 87.3% was calculated for the NDV NP_ct_ recombinant antigen using the HI as the standard assay. Inter-rater agreement between the NP_ct_ ELISA results and HI titers (kappa = 0.53 [CI 0.438–0.622]) was slightly better compared to full length NP and the commercial ELISA. None of the field sera scored positive for APMV-8 NP_ct_ specific antibodies (Table 2).

## Discussion

We successfully constructed, bacterially expressed, and purified full-length and truncated versions of the protein products of the NP and P genes of the APMV-1 strain LaSota. Truncations were chosen so as to express less conserved (C-terminal part of NP) or unique (P_u_, V_u_) amino acid sequence stretches (Fig. 1). In order to improve the immunological reactivity of the rather small peptides (ranging from 11.7 to 27.6 kD) they were expressed as fusion proteins of the human C4 binding protein heptamerization domain [42]. Analysis of refolded proteins revealed all of the C4BP-tagged proteins formed multimers with an approximately sevenfold molecular weight compared to monomers. This indicates that the C4BP domain is potent to promote protein multimerization. Part of full-length NP expressed without a C4BP tag was monomeric but spontaneous formation of dimers, trimers and multimers under non-denaturing PAGE conditions was observed as well (Fig. 2). Spontaneous self-assembly/multimerization of full length NP proteins of negative-stranded RNA viruses with helical nucleocapsids had been reported before [17, 49]. Recombinant proteins were purified from bacterial inclusion bodies, solubilized, and refolded in vitro, and their antigenic authenticity was confirmed using a chicken immune serum obtained after immunization with live-attenuated NDV vaccine (Fig. 2). Results were encouraging to use the recombinant proteins as antigens for the detection of APMV1 specific antibodies.

**Fig. 6:**
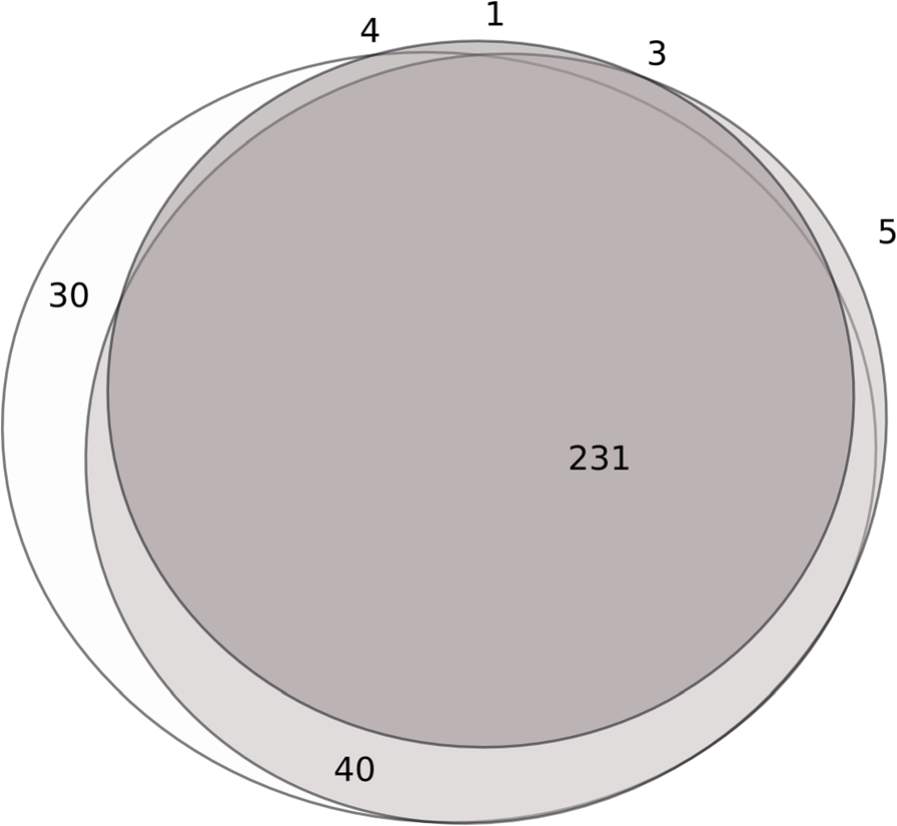
Venn diagram [54] of detection of NDV NP-specific antibodies by commercial indirect ELISA (white ellipsis), recombinant indirect ELISA using full-length NP (light grey ellipsis) or the truncated C-terminal fragment of NP (LS-NP_ct_, dark grey circle) in chicken and turkey field sera. Numbers indicate positive sera in a given subset except for the centre of the circle where also congruently negative sera have been added

Currently, serological testing for avian avulavirus antibodies, in particular of APMV-1, as employed, e.g., for surveillance purposes, is traditionally based on HI assay or indirect ELISAs using whole virus antigen [2] or full-length NP protein [14–16, 35, 50]. Although ELISAs are widely used for screening purposes, little is known about their specificity regarding cross reacting antibodies induced after infection with other APMV serotypes. In this study, when testing hyperimmune sera raised in chickens against nine different APMV serotypes in the different ELISAs, cross reactions were observed for the commercial ELISA and also for the recombinant ELISAs based on full-length NP and the unique P proteins (Fig. 3a and c). As hypothesized, specificity increased when using a less conserved C-terminal fragment of the NDV or the APMV-8 NP proteins (Fig. 3b). Our results confirmed that the C-terminal NP fragment holds potential as a highly serotype-specific diagnostic antigen. However, compared to full-length NP, the number of antigenic sites is substantially reduced. This fact may be at the basis of the lower sensitivity of NP_ct_ driven indirect ELISAs (compared to commercial and full-length NP ELISAs) as observed in this study with field sera (Fig. 6, Table 2). This may limit the general use of NP_ct_ fragments if a screening assay of high sensitivity is required. However, higher sensitivity, i.e. the use of complete virus or full-length NP protein as diagnostic antigens, is achieved only at the expense of a loss of specificity as our data on APMV serotype-specific hyperimmune sera showed. The NP_ct_ fragment, in contrast, provides high serotype specificity similar to that of HI assays.

To evaluate the V protein as a target antigen in the NDV DIVA strategy, sera of chickens immunized with live attenuated NDV clone 30 vaccine and subsequently challenged with virulent Herts33 were used. Notably, the complete V protein is generated from the P gene via post-transcriptional mRNA editing, and thus P and V proteins possess an identical portion at their N-terminus. To eradicate the cross reaction between P and V proteins, the distinctive C-terminal fragment of the V protein (V_u_) was expressed as target antigen in the iELISA. As a result, only a few of the animals (2/10) seroconverted against the V protein after immunization with live attenuated vaccine, but when challenged with a virulent virus this number of animals rose to seven out of ten (Table 1). In conjunction with the observation that non-vaccinated control animals seroconverted against V_u_ after challenge with the velogenic NDV Herts strain, these results indicated that V protein may be more immunogenic during infection with virulent strains. The data gave evidence that, on a flock level, (i) seroconversion to V after application of live-attenuated vaccine was incomplete and (ii) V-specific antibody titers declined within a few weeks to below detection limits. This significantly limits the use of a V protein based DIVA strategy for NDV.

## Conclusions

In this study, a newly configured bacterial expression system featuring co-translationally monobiotinylated fusion proteins of small protein fragments of NDV NP and P (V) gene products fused with the heptamerization domain of human C4BP yielded immune reactive products. Utility of the recombinant proteins as serodiagnostic antigens was demonstrated in indirect ELISA assays: (i) the NP_ct_ protein fragments of NDV and APMV-8 enabled a serotype specific diagnosis by ELISA and might be exploited as alternative, highly serotype-specific diagnostic tools for classical HI; (ii) the NDV V protein is able to induce some weak humoral immune response after immunization with an attenuated NDV vaccine strain. However, V-specific seroconversion within an experimentally infected flock was incomplete, and titers in individual bird transient. Therefore, a V protein-based serological DIVA strategy to distinguish infected from vaccinated (inactivated vaccines) birds is not feasible; (iii) the full-length NP protein as the ELISA antigen possessed comparable diagnostic features compared to a whole virus antigen based commercial ELISA, but cross reactivity among APMV serotypes may blur specificity.

## Additional file

Additional file 1: Cut-off values of indirect ELISAs based on recombinant avian paramyxovirus NP and P gene products. Provides data that elucidate the fixing of a threshold for indirect ELISA (DOCX 12 kb)
Additional file 2: Identity and similarity of the C-terminus of the nucleocapsid protein of avian paramyxoviruses. Provides a similarity matrix of the C-terminus of the NP protein of avian parmayxoviruses. Protein sequences were retrieved from GenBank, aligned with MAFFT [51], trimmed with Jalview [52] and analysed using MatGat [53]. Alignment started from Identity values upper right, similarity lower left matrix. Values greater 65% are greyed.(DOCX 20 kb)

## Abbreviations

APMV: Avian paramyxovirus
C4BP: C4 binding protein
DIVA: Differentiating infected from vaccinated animals
LS: LaSota strain of NDV
NDV: Newcastle disease virus (APMV-1)
NP: Nulceocapsid protein
NPct: C-terminal fragment of NP
TBST: Tris-buffered saline supplemented with 0.05% (*v*/v) Tween
U: Unique parts of phosphor- and V proteins
