# Supplementary material for: Engineered recombinant protein products of the avian paramyxovirus type-1 nucleocapsid and phosphoprotein genes for serological diagnosis
Source: Virol J. 2018 Jan 11;15:8. doi: 10.1186/s12985-018-0924-8 (PMC5765633; doi:10.1186/s12985-018-0924-8)
Supplement: Additional file 1: — Cut-off values of indirect ELISAs based on recombinant avian paramyxovirus NP and P gene products. Provides data that elucidate the fixing of a threshold for indirect ELISA (DOCX 12 kb) [file 12985_2018_924_MOESM1_ESM.docx]

**Additional file 1: Table S2.** Cut-off values of indirect ELISAs based on recombinant avian paramyxovirus NP and P gene products.

| Serum samples | Values | Recombinant antigens | | | | |
| --- | --- | --- | --- | --- | --- | --- |
|  |  | NP | P_u_ | V_u_ | NP_ct_ | APMV-8 NP_ct_ |
| SPF chicken sera ([S-N]/[P-N] values) | Mean | 0.076 | 0.016 | 0.03 | 0.13 | 0.166 |
| N=51 | SD | 0.09 | 0.059 | 0.027 | 0.161 | 0.149 |
| Cut off | Xq + 2SD | 0.257 | 0.134 | 0.083 | 0.451 | 0.465 |

Viruses used for production of recombinant proteins:

NP, P_u_, V_u_, NP_ct_ – LaSota strain of APMV-1

APMV-8 NP_ct_ – APMV-8/goose/2010
