# Supplementary material for: Engineered recombinant protein products of the avian paramyxovirus type-1 nucleocapsid and phosphoprotein genes for serological diagnosis
Source: Virol J. 2018 Jan 11;15:8. doi: 10.1186/s12985-018-0924-8 (PMC5765633; doi:10.1186/s12985-018-0924-8)
Supplement: Additional file 2: — Identity and similarity of the C-terminus of the nucleocapsid protein of avian paramyxoviruses. Provides a similarity matrix of the C-terminus of the NP protein of avian parmayxoviruses. Protein sequences were retrieved from GenBank, aligned with MAFFT [51], trimmed with Jalview [52] and analysed using MatGat [53]. Alignment started from Identity values upper right, similarity lower left matrix. Values greater 65% are greyed.(DOCX 20 kb) [file 12985_2018_924_MOESM2_ESM.docx]

**Additional file 2: Table S1.** Identity and similarity of the C-terminus of the nucleocapsid protein of avian paramyxoviruses.

| Strain designation |  | 1 | 2 | 3 | 4 | 5 | 6 | 7 | 8 | 9 | 10 | 11 | 12 | 13 | 14 | 15 | 16 | 17 |
| --- | --- | --- | --- | --- | --- | --- | --- | --- | --- | --- | --- | --- | --- | --- | --- | --- | --- | --- |
| APMV-1/vac/Lasota_EF442113/59-157 | 1 |  | 68 | 71 | 19 | 17 | 23 | 20 | 20 | 19 | 18 | 33 | 17 | 15 | 34 | 27 | 22 | 22 |
| APMV-1/Duck/Germany/49/1999_DQ097393/59-157 | 2 | 79 |  | 86 | 22 | 17 | 27 | 24 | 25 | 22 | 18 | 37 | 20 | 19 | 36 | 28 | 17 | 24 |
| APMV-1/Teal/France/100011/2010_JQ013039/59-157 | 3 | 79 | 91 |  | 21 | 17 | 27 | 22 | 28 | 23 | 21 | 34 | 24 | 22 | 33 | 26 | 18 | 26 |
| APMV-2/Chicken/England/7702/06_HM159993/59-123 | 4 | 30 | 35 | 32 |  | 21 | 26 | 26 | 35 | 22 | 28 | 20 | 30 | 23 | 17 | 18 | 28 | 28 |
| APMV-3/Parakeet/Netherland/449/75_EU403085/59-125 | 5 | 26 | 25 | 30 | 42 |  | 19 | 21 | 16 | 19 | 19 | 13 | 23 | 19 | 21 | 21 | 25 | 22 |
| APMV-4/Duck/Delaware/549227/2010_NC_019531/57-123 | 6 | 31 | 35 | 34 | 45 | 43 |  | 22 | 20 | 20 | 25 | 19 | 17 | 23 | 26 | 21 | 23 | 21 |
| APMV-5/Buderigar/Kunitachi/74_GU206351/59-131 | 7 | 30 | 33 | 30 | 45 | 43 | 37 |  | 33 | 23 | 26 | 16 | 24 | 27 | 16 | 19 | 32 | 34 |
| APMV-6/Duck/Taiwan/Y1/98_ENC_003043/59-131 | 8 | 32 | 35 | 38 | 53 | 34 | 37 | 52 |  | 31 | 27 | 22 | 30 | 27 | 24 | 23 | 32 | 26 |
| APMV-7/Dove/Tennessee/4/75_FJ231524/59-129 | 9 | 26 | 34 | 30 | 44 | 41 | 38 | 36 | 45 |  | 30 | 16 | 23 | 26 | 18 | 18 | 29 | 23 |
| APMV-8/Goose/Delaware/1053/76_FJ619036/59-127 | 10 | 29 | 33 | 35 | 49 | 44 | 41 | 44 | 49 | 41 |  | 20 | 28 | 26 | 19 | 19 | 23 | 30 |
| APMV-9/Duck/New York/59-157 | 11 | 50 | 54 | 52 | 32 | 26 | 29 | 29 | 32 | 27 | 29 |  | 22 | 19 | 26 | 27 | 20 | 17 |
| APMV-10/Penguin/Falkland Islands/437/2007_HM755887 | 12 | 25 | 28 | 30 | 49 | 42 | 36 | 41 | 43 | 35 | 52 | 29 |  | 29 | 18 | 16 | 27 | 38 |
| APMV-11/Common_snipe/France/100212/2010_JQ886184/59-121 | 13 | 26 | 27 | 30 | 43 | 43 | 40 | 43 | 44 | 41 | 39 | 32 | 42 |  | 19 | 17 | 32 | 25 |
| APMV-12/Wigeon/Italy/3920_1/2005_KC333050/59-161 | 14 | 49 | 51 | 51 | 28 | 34 | 33 | 27 | 36 | 26 | 35 | 46 | 31 | 32 |  | 44 | 18 | 21 |
| APMV-13/Goose/Shimane/67/2000_NC_030231 | 15 | 50 | 48 | 45 | 25 | 32 | 34 | 35 | 34 | 29 | 34 | 52 | 29 | 28 | 65 |  | 18 | 19 |
| APMV-14/Duck/Japan/11OG0352/2011_KX258200 | 16 | 31 | 28 | 28 | 37 | 45 | 47 | 45 | 51 | 42 | 49 | 32 | 38 | 51 | 31 | 37 |  | 35 |
| APMV-15/Calidris_fuscicollis/Brazil/RS-1177/2012_NC_034968 | 17 | 29 | 31 | 33 | 49 | 45 | 39 | 49 | 41 | 41 | 49 | 29 | 51 | 43 | 31 | 36 | 48 |  |

Protein sequences were retrieved from GenBank, aligned with MAFFT [52], trimmed with Jalview [53] and analysed using MatGat [54]. Alignment started from Identity values upper right, similarity lower left matrix. Values greater 65% are greyed.
